# Construction and Identification of New Molecular Markers of Triple-Negative Breast Cancer Stem Cells

**DOI:** 10.3389/fonc.2021.647291

**Published:** 2021-05-28

**Authors:** Tingting Liu, Hongyue Wang, Zhiyong Liu, Jing Zhang, Yan Liu, Lin Zhang, Chunhui Zheng, Fei Liu, Chuanqiang Hou, Baojiang Li

**Affiliations:** ^1^Department of Breast Surgery, Breast Cancer Center, Tai'an Central Hospital, Tai'an, China; ^2^Department of Central Sterile Supply, Tai'an Central Hospital, Tai'an, China; ^3^Department of Ultrasonic Diagnosis, Breast Cancer Center, Tai'an Central Hospital, Tai'an, China; ^4^Department of Oncology Surgery, Weifang People's Hospital, Weifang, China

**Keywords:** triple-negative breast cancer, cancer stem cell, marker, phage, identification

## Abstract

**Objective:** We screened the TNBC stem cells using phage display (PD) and acquired the specific binding clones; and then the positive phage DNAs were amplified and extracted, synthesized with specific polypeptides, and labeled with fluorescein isothiocyanate (FITC). Finally, we identified the specificity of the polypeptides *in vitro* and *in vivo*.

**Methods:** Human breast cancer cell line MDA-MB-231 and human mammary gland cell line hs578bst were chosen in our study, and MDA-MB-231 breast cancer stem cells (BCSCs) were cultured and identified by flow cytometry. The phage peptide library was screened using MDA-MB-231 BCSCs, the positive phage clones were identified by ELISA, and the DNA of the positive phages was extracted and sent to a biotechnology company for sequencing. According to the sequencing results, a specific polypeptide was synthesized and labeled with FITC. In the end, the specificity of a polypeptide to BCSCs was identified *in vivo* and *in vitro*.

**Results:** The MDA-MB-231 BCSCs were cultured and enriched with the “serum and serum-free alternate” method. The BCSCs were found to have characteristics of CD44^+^/CD24^−/*low*^ epithelial surface antigen (ESA) and ALDH^+^ with flow cytometry. The phage was enriched to 200-fold after three rounds of screening for MDA-MB-231 BCSCs. The positive phages were sequenced; then a polypeptide named M58 was synthesized according to sequencing results. Polypeptide M58 has a specific affinity to MDA-MB-231 BCSCs *in vivo* and *in vitro*.

**Conclusion:** Specific polypeptides binding to MDA-MB-231 BCSCs were screened out by PD screening method, which laid a theoretical foundation for the targeted therapy and further research of BCSCs.

## Introduction

Triple-negative breast cancer (TNBC) accounts for approximately 15–20% of all breast cancers and characterized by the lack of expression of estrogen receptor (ER), progesterone receptor (PR), and human epidermal growth factor receptor 2 (HER2) (1). Because of the lack of specific therapeutic targets for the type of breast cancer, there is currently no available efficient treatment for TNBC. The majority of TNBC patients are at a higher risk of tumor recurrence and metastasis (2), and more efforts are needed to find new therapeutic targets and methods for this type of cancer.

In human cancer, including breast cancer, there is a small subset called cancer stem cells (CSCs), which possess stemness properties and are capable of self-renewal, differentiation, and tumor initiation and growth (3). Cancer stem cells also contribute to tumor recurrence, due to their inherent distinct biological properties, such as resistance to chemotherapy and radiotherapy (4). The earliest CSCs isolated and characterized in solid tumors were from breast cancer. These breast cancer stem cells (BCSCs) were identified by the feature of cell surface marker CD44^high^CD24^low^ and aldehyde dehydrogenase (ALDH) enzymatic activity (5, 6). A few of BCSCs can lead to xenograft tumor formation in immunodeficient non-obese diabetic (NOD)/severe combined immunodeficiency (SCID) mice (7). Traditional chemotherapy and radiotherapy can only kill ordinary breast cancer cells and are less effective to BCSCs, leading to recurrence or metastasis in breast cancer. Only targeted elimination of BCSCs is the key at which breast cancer may be cured completely.

Phage display (PD) is a technology based on the presentation of functional peptides on the surface of bacteriophages and was invented by George Smith in 1985. Ever since it appeared, PD technique has revolutionized several biological fields because of its obvious power for the production of many kinds of proteins and its relatively fast speed for the isolation of biological compounds (8, 9). Until now, PD technology is used in a wide range of fields, such as oncology, cell biology, immunology, pharmacology, and drug discovery and delivery. In breast cancer, an aFGF-binding peptide called AP8 was shown to interact with FGFRs, as both breast cancer and vascular endothelial cells were observed to be arrested in the G0/G1 stage (10). Novel peptides that had been screened from a peptide library were shown to bind to CD44 with high affinity (11). PD technology is a mature type of technology for the screening of tumor-specific peptides (12); however, the application of BCSC-specific peptides has been seldom reported.

## Materials and Methods

### Reagents and Cell Lines

The bacteriophage random 12-peptide library kit (Ph.D.™-12 Phage Display Peptide Library) was purchased from New England BioLabs, Inc., Ipswich, MA, USA. Flow cytometry (FCM) antibody, fluorescein isothiocyanate (FITC) anti-human CD44 antibody, phycoerythrin (PE) anti-human CD24 antibody, and Alexa Fluor647 anti-human CD326 antibody were purchased from BioLegend, Inc., San Diego, CA, USA; EGF, bFGF, and B27 growth factors were purchased from Sigma-Aldrich (Merck KGaA, Darmstadt, Germany). The human breast cancer cell line MDA-MB-231 and the human mammary gland cell line hs578bst were preserved in our laboratory.

### Enrichment and Identification of Breast Cancer Stem Cells

Routine cell culture was mixed with Dulbecco's modified Eagle's medium (DMEM) containing 10% fetal bovine serum (FBS), 100 U/ml of penicillin, and 100 U/ml of streptomycin. Stem cells were enriched in serum-free medium supplemented with EGF, bFGF, and B27 growth factors. Centrifugation was conducted to change the medium every 2–3 days. After 1 week of serum-free culture, the medium was changed to medium with 10% FBS for one passage to remove any dead cells. The cells were cultured alternately with serum and serum-free culture medium to maximize BCSCs. After that, CD44^+^/CD24^−/*low*^ cell group was sorted using a flow cytometer, ALDH^+^ was detected, and the microspheres were observed under a microscope.

### Phage Random Peptide Screening for Breast Cancer Stem Cells

The hs578bst cells, breast cancer cells, and enriched BCSCs were seeded in polylysine-coated petri dishes with serum-free DMEM for 2 h and blocked with 0.5% bovine serum albumin (BSA) for 1 h. The PD peptide library was added to the dish coated with the hs578bst cells with 10^11^ pfu/titer, then they were cultured at 37°C for 1 h, and the supernatant was transferred to the negative selection cells. The above two steps were repeated three times to complete three negative and one positive selection. The cells were washed with 0.1% Tris-buffered saline with Tween (TBST) three times for 1 min each time after the incubation, with care taken to change the paper every time to avoid cross contamination. The phages were removed by washing with 1 ml of 0.2 M Glycine-HCl (pH 2.2) buffer. The cell supernatant was collected in a centrifuge tube after incubation for 10 min and neutralized with 150 μl of Tris-HCl (pH 9.1). The product was amplified and titrated for the next round of selection. In the following round of selection, conditions were not changed except that the total amount of each initial phage was 1 × 10^11^, the time for positive selection was 30 min, and 0.2% (v/v) Tween-20 was used for washing. In the third round of screening, the positive selection time was 15 min, and 0.3% (v/v) Tween-20 was used for washing.

### ELISA Identification for Positive Phages

The enriched BCSCs were seeded in 96-well plates at a number of 10^4^ per well with serum-free DMEM for 2 h after adherence. The cells were fixed with 4% paraformaldehyde for 15 min and then washed with phosphate-buffered saline (PBS). The cells were treated with 0.1% Triton X-100 for 10 min and washed with PBST-0.05% three times. After 1 h of blocking with 2% PBS-BSA, the cells were incubated with amplified monoclonal phage for 2 h and washed three times with PBST-0.05%. After incubation with horseradish peroxidase (HRP)-anti-M13 antibody (1:5,000 with 2% PBS-BSA) for 1 h, cells were washed with PBST-0.05% three times. HCl was added to terminate the TMB chromogenic reaction, and the absorbance was read at 450 nm using a microplate reader. A phage plaque was randomly selected as a control, and the value of OD phage clone/OD control >2 was regarded as positive. Normal breast cells, and breast cancer and enriched BCSCs were seeded in a 24-well plate at 10^5^ cells per well, and the same process was repeated except that TMB was replaced with a DAB HRP chromogenic kit and HCl was replaced with distilled water after a 10-min incubation. Cells were counterstained with Mayer's hematoxylin solution and observed under a microscope.

### DNA Extraction of Positive Phage and Sequencing

The single colonies of *Escherichia coli* ER2738 were inoculated into 20 ml of lysogeny broth (LB) medium and shaken and cultured to early logarithmic growth phase. The KL-6 stock solution with a total of 10 μl of positive phage clone was added to the ER2738 solution, which was liquefied and centrifuged at 37°C and 250 rpm for 3.5 h. After centrifugation at 10,000 rpm for 5 min, the supernatant was added to 1/6 volume of 20% PEG/NaCl to precipitate at room temperature for 1 h and centrifuged at 10,000 rpm for 10 min. The supernatant was removed, and the precipitation was resuspended with 1 ml of TBS and stored at 4°C. During the course of plaque amplification, 500 μl of phage-containing supernatant was transferred to a new Eppendorf (EP) tube after the first centrifugation. A total of 200 μl of PEG/NaCl was added, and the mixture was inverted and mixed well. Subsequently, the mixture was allowed to rest at room temperature for 10 min. Afterward, the sample was centrifuged for 10 min, and the supernatant was discarded. The sample was centrifuged briefly again, and the remaining supernatant was carefully aspirated. The pellet was completely resuspended in 100 μl of iodide buffer; 250 μl of ethanol was added and incubated at room temperature for 10 min. Single-stranded phage DNA was incubated and precipitated at room temperature for a short time, while most of phage proteins remained in the solution. Then the sample was centrifuged for 10 min, and the supernatant was discarded after incubation. The precipitate was washed with 70% ethanol and briefly vacuum dried. The pellet was resuspended in 30 μl of TE, and the resulting suspension was used as the template solution for sequencing. Sequencing primer is−96 gIII 5′-HOCCC TCA TAG TTA GCG TAA CG-3′.

### The Specificity Identification of Polypeptide *in vitro*

The polypeptides were synthesized according to the sequencing result and labeled with FITC. The breast cancer cells and BCSCs were incubated with the polypeptides labeled with FITC. Then, the distribution of the FITC-labeled polypeptides was observed in different cells, and the images were captured.

### The Specificity Identification of Polypeptide *in vivo*

We chose 20 female nude BALB/c mice (6–8 weeks) to establish an animal model after we acquired the approval of Ethics Committee of Shandong Cancer Hospital and Institute. The *ad libitum*-fed mice were kept in specific pathogen-free (SPF) environment of 20-Pa pressure difference, 45% humidity, 22°C temperature, and a 14/10-h light/dark cycle. Firstly, we centrifuged MDA-MB-231 stem cells, adjusted the concentration of BCSCs to 1 × 10^5^/ml, and then implanted them subcutaneously in the armpit of the right lower limb or the right breast pad or intravenously into the tail vein of nude BALB/c mice. Secondly, we measured the tumor size and randomly divided nude mice into two equal groups, named group M58 and group M0. Lastly, we injected the polypeptide into the vein of nude mice and dissected them to observe the polypeptide distribution in liver tissue with the control tissue (of the liver). All animal experiments were performed in accordance with guidelines for proper conduct of animal experiments.

## Results

### The Culture and Enrichment of Breast Cancer Stem Cells

**Figure 1:**
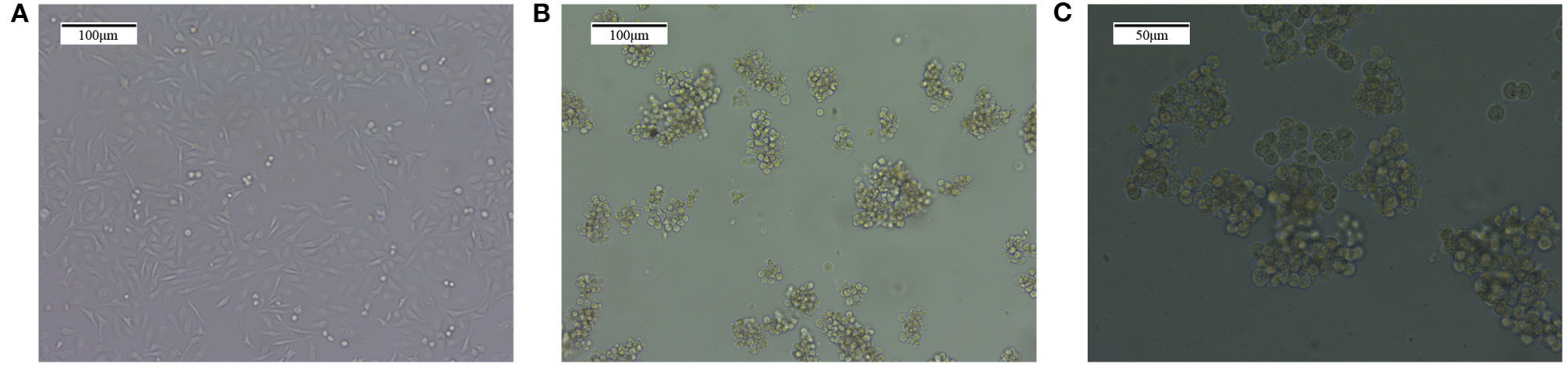
The enrichment of MDA-MB-231 breast cancer stem cells with the method of “serum and serum-free alternation.” **(A)** The ordinary MDA-MB-231 breast cancer cells. **(B)** The MDA-MB-231 cancer stem cell microspheres formed after “serum and serum-free alternation” culture for 30 days, which are balloon shaped and suspended in the culture solution. Magnification, 10×. **(C)** The MDA-MB-231 cancer stem cell microspheres. Magnification, 10×.

The common MDA-MB-231 breast cancer cells were cultured for 30 days with the method of “serum and serum-free alternation.” Afterward, the BCSC microspheres were suspended in the culture medium under the microscope. They looked round and bright and are balloon shaped, and the volume and number of stem cells increased following the culture time extension, as shown in Figure 1.

### The Identification of Breast Cancer Stem Cells With Flow Cytometry

**Figure 2:**
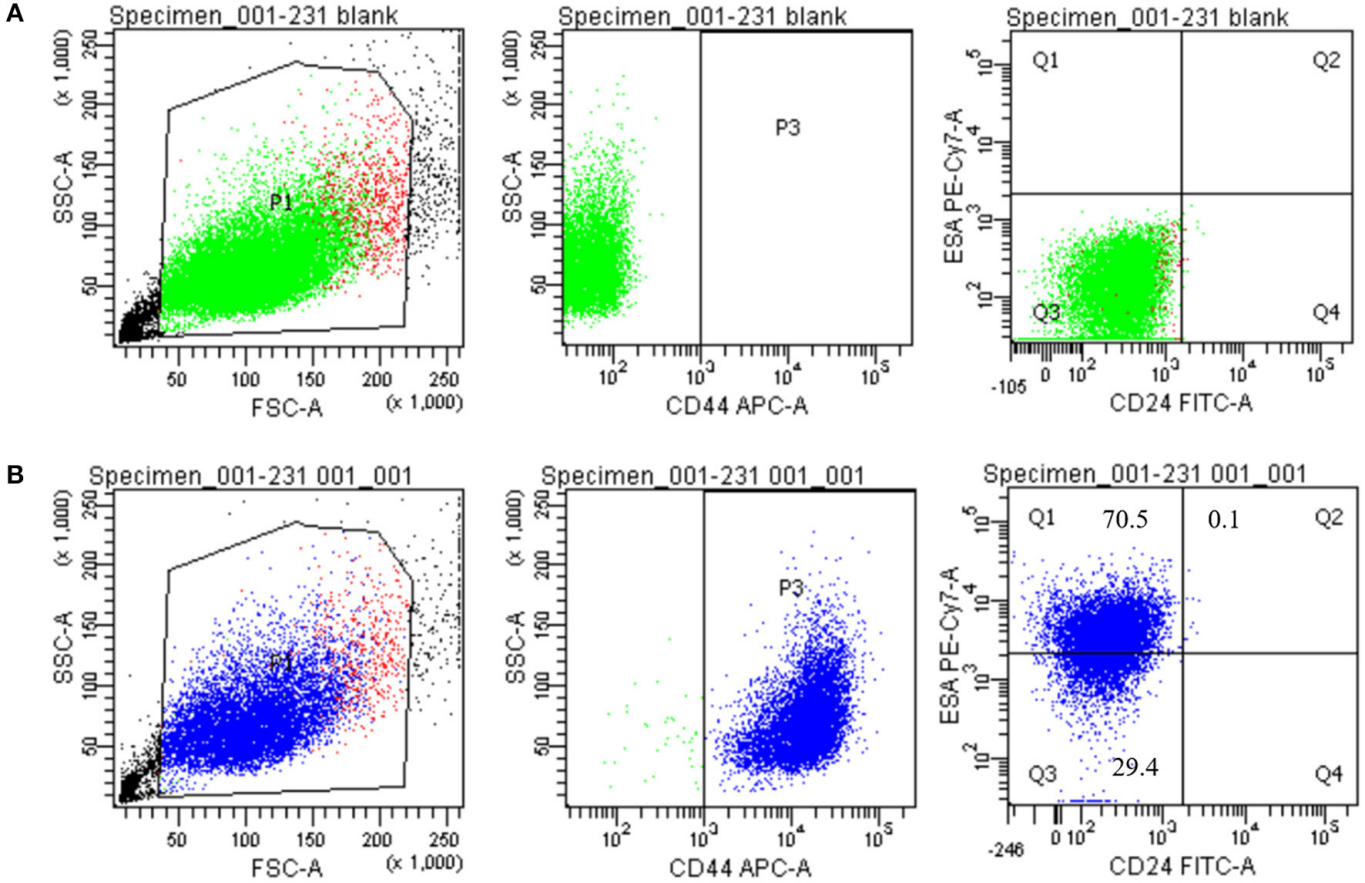
The identification results of CD44^+^/CD24^−/*low*^ with flow cytometry. **(A)** The proportion of CD44^+^/CD24^−/*low*^ for ordinary MDA-MB-231 was nearly zero. **(B)** The proportion of CD44^+^/CD24^−/*low*^ for MDA-MB-231 breast cancer stem cell was 70.5%. ESA APC, epithelial surface antigen allophycocyanin; FSC, forward scatter; P1, breast cancer stem cell; P2, CD44^+^ cells; PE, phycoerythrin; SSC, side scatter.

**Figure 3:**
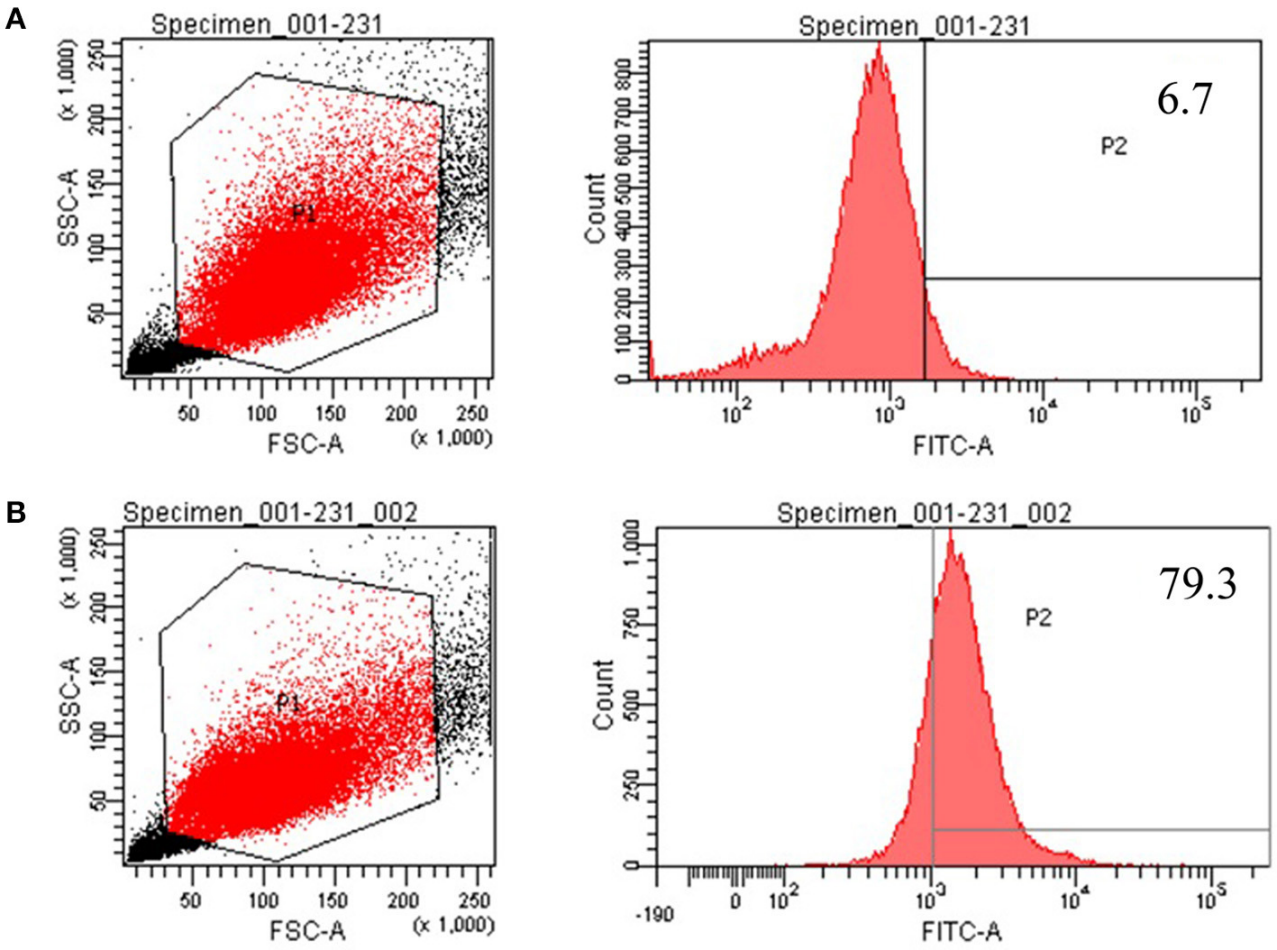
The identification results of ALDH^+^ with flow cytometry. **(A)** The proportion of ALDH^+^ for ordinary MDA-MB-231 was 6.7%. **(B)** The proportion of ALDH^+^ for MDA-MB-231 breast cancer stem cell was 79.3%.

We chose CD44^+^/CD24^−/*low*^ and ALDH^+^ as the biomarker of MDA-MB-231 BCSCs compared with the common MDA-MB-231 breast cancer cells. As Figure 2 shows, the proportion of CD44^+^/CD24^−/*low*^ for MDA-MB-231 BCSCs was 70.5%, while the proportion for common breast cancer cells was nearly zero. Simultaneously, the proportion of ALDH^+^ for MDA-MB-231 BCSCs was 79.3%, while the proportion for common breast cancer cells was 6.7%, as shown in Figure 3.

### Screening of Phage Specificity for Breast Cancer Stem Cells

The phages were enriched nearly 200 times after three rounds of screening, as shown in Table 1, and indicated that the phage may specifically bind to MDA-MB-231 BCSCs.

**Table 1:** Results of three rounds of phage screening in MDA-MB-231 breast cancer stem cells.

**Round**	**Initial phage volume (pfu/ml)**	**Elution phage volume (pfu/ml)**	**Enrichment rate (%)**
First round	2.00 × 10^10^	4.20 × 10^2^	2.1 × 10^−8^
Second round	2.00 × 10^10^	7.40 × 10^3^	3.7 × 10^−7^
Third round	2.00 × 10^10^	8.40 × 10^4^	4.2 × 10^−6^

### DNA Sequence of Positive Phages

Eight positive phages were collected by M13 phage library isolation kit with ELISA identification. They were named M1–M8, and the Amino acid sequences are shown in Table 2. The sequence of TMHYKGTAASES appeared twice, which was selected to synthesize the polypeptide named M58 for subsequent experiments, and the negative peptide sequences were NHKTINYQNDAT and named M0 as control.

**Table 2:** Results of DNA sequences for positive phages.

**Name**	**Amino acid sequence**
M1	LYAVDLSPKSRY
M2	HLAVRPISTNSR
M3	HLAVRPISTNSR
M4	TNSFHAIAGYQS
M5, M8	**TMHYKGTAASES**
M6	KLTALVTTWPWT
M7	YSDGVRAPRTVE

The complete sequence results of M58 are as follows:

3′-TCCCGACGTTAGTAAATGAATTTTCTGTATGGGATTTTGCTAAACAACTTTCAACAGTTTCGGCCGAACCTCCACCCGA CTC AGA AGC CGC CGT CCC CTT ATA ATG CAT CGT AGAGTGAGAATAGAAAGGTACCACTAAAGG AATTGCGAATAAAAATAGTC CCCCAAA-5′

5′-ACG ATG CAT TAT AAG GGG ACG GCG GCT TCT GAG TCG-3′

T M H Y K G T A A S E S.

The complete sequence results of M0 are as follows:

3′-TCCGACGTTAGTAAATGAATTTTCTGTATGGGATTTTGCTAAACAACTTTCAACAGTTTCGGCCGAACCTCCACCAGTCGCATCATTCTGATAATTAATCGTCTTATGATTAGAGTGAGAATAGAAAGGTACCACTAAAGGAATTGCGAATAACAAATGCCATCCGACTGTTTTGCCCTCCTCAATACGTGAAGCTGCAGCCCTCCTCTTATTGTTGAGCTCTATCACAGAGGTGTTAGTCGCGTTAACGCTACCATGTATCTCTTGGTTAGAGCAGATGTAAGAGGAAAAAAAGTTCCGTGCGTATT-5′

5′ AAT CAT AAG ACG ATT AAT TAT CAG AAT GAT GCG ACT 3′

N H K T I N Y Q N D A T.

### The Specificity Identification of Polypeptide *in vitro*

**Figure 4:**
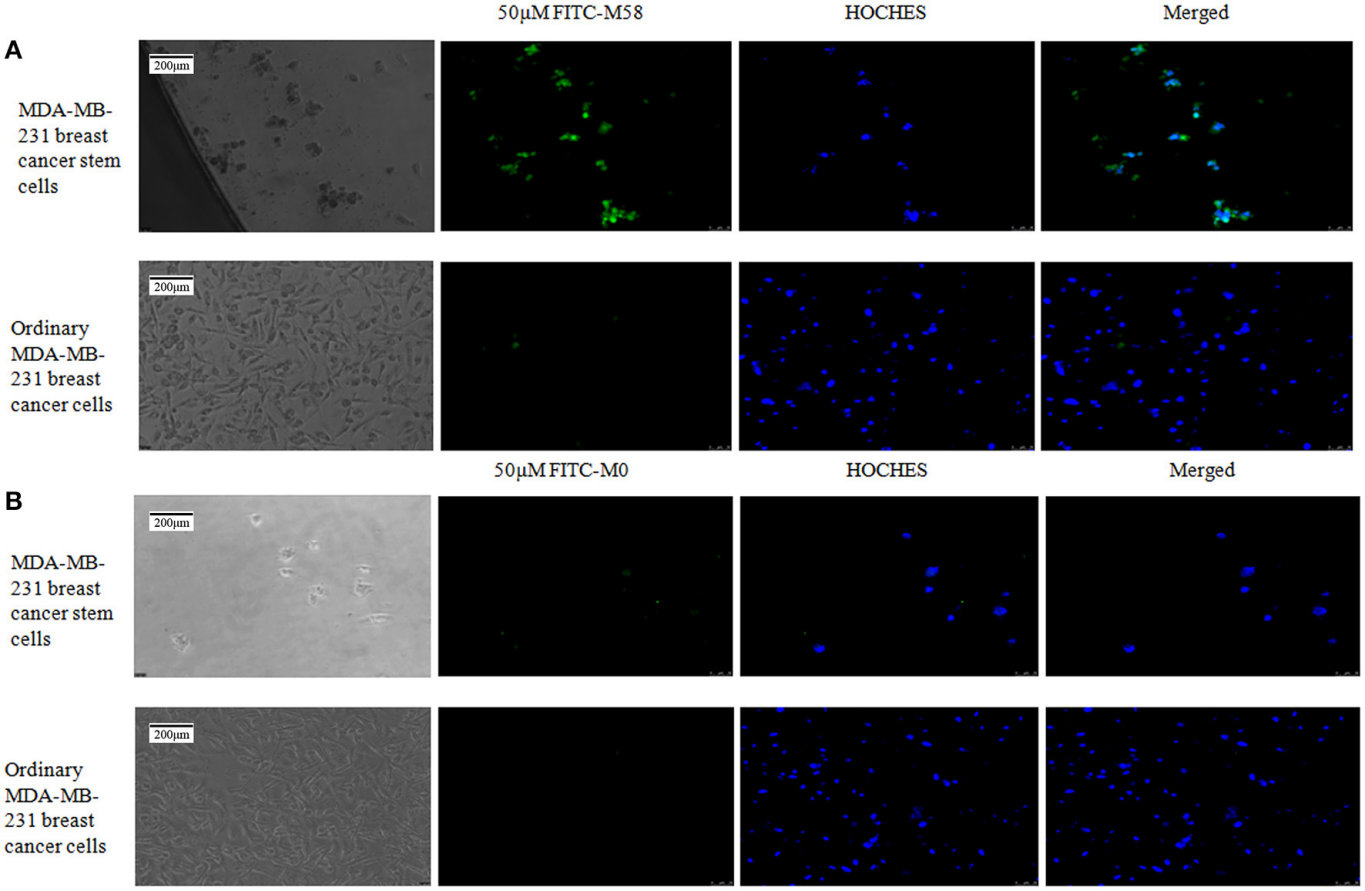
Binding status of polypeptide M58 to MDA-MB-231 breast cancer stem cells and ordinary MDA-MB-231 breast cancer cells. **(A)** The fluorescein isothiocyanate (FITC)-labeled M58 was incubated with MDA-MB-231 breast cancer stem cells and ordinary MDA-MB-231 breast cancer cells; polypeptide M58 labeled with FITC was observed to specifically bind to MDA-MB-231 breast cancer stem cells, but not to ordinary MDA-MB-231 breast cancer cells. **(B)** The FITC-labeled control polypeptide M0 was incubated with MDA-MB-231 breast cancer stem cells and ordinary MDA-MB-231 breast cancer cells; the control polypeptide M0 labeled with FITC was observed to neither bind to MDA-MB-231 breast cancer stem cells nor to ordinary MDA-MB-231 breast cancer cells.

Polypeptide M58 labeled with FITC was able to specifically bind to the MDA-MD-231 stem cells, while it could not bind to the common MDA-MD-231 cells, as shown in Figure 4, whereas the control peptide M0 labeled with FITC was not able to bind to the MDA-MD-231 stem cells in either of the common cells.

### The Specificity Identification of Polypeptide *in vivo*

**Figure 5:**
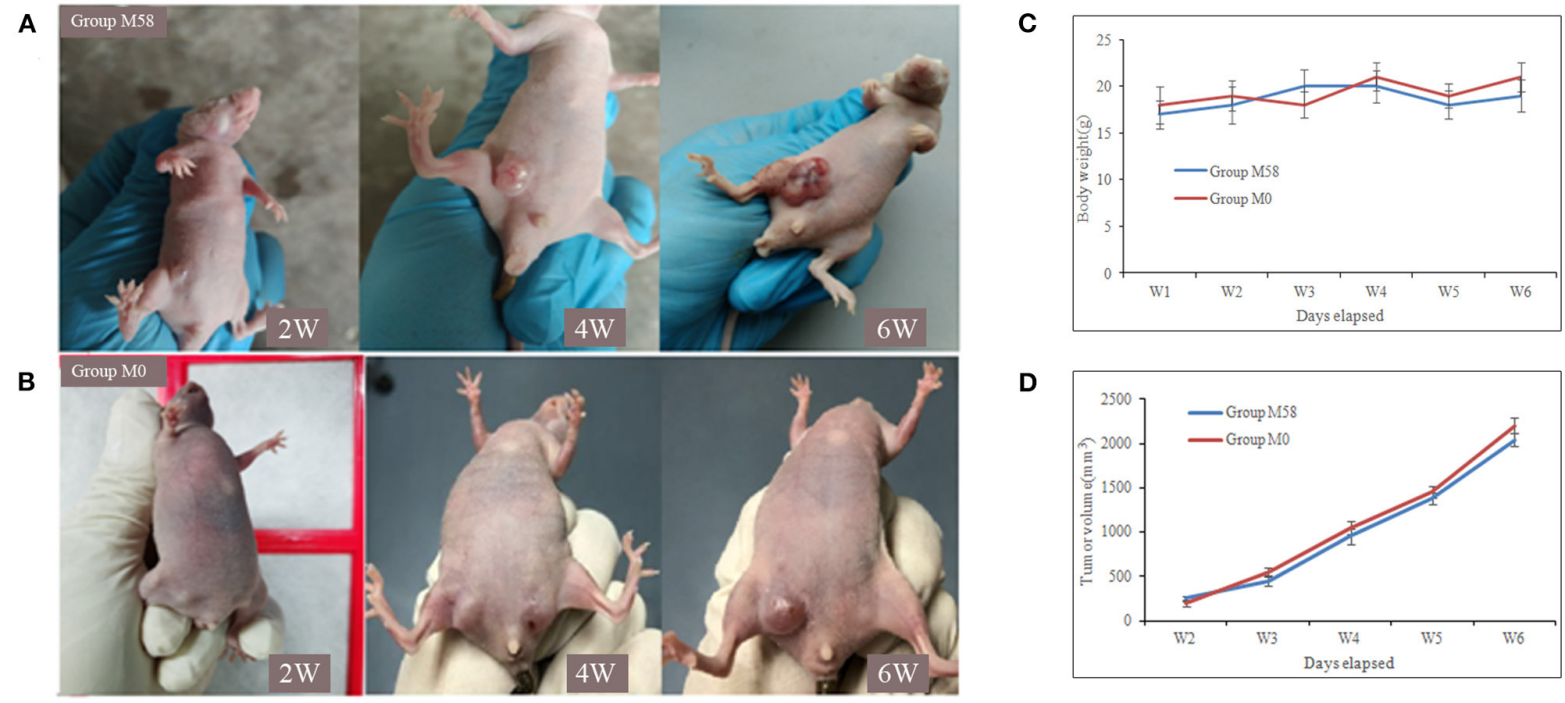
Tumor formation of nude mice after breast cancer stem cell (BCSC) inoculation. **(A,B)** The MDA-MB-231 BCSCs were injected subcutaneously in the armpit of the right lower limb, and the tumor formed and increased gradually. The nude mice of group M58 were used for the specificity identification of polypeptide M58 and group M0 for control peptide M0. **(C)** The body weights of the mice in two groups and the difference between them were not statistically significant. **(D)** Tumor volume was measured using a caliper and calculated as (width^2^ × length)/2; the tumor volume of the two groups was not statistically significant.

**Figure 6:**
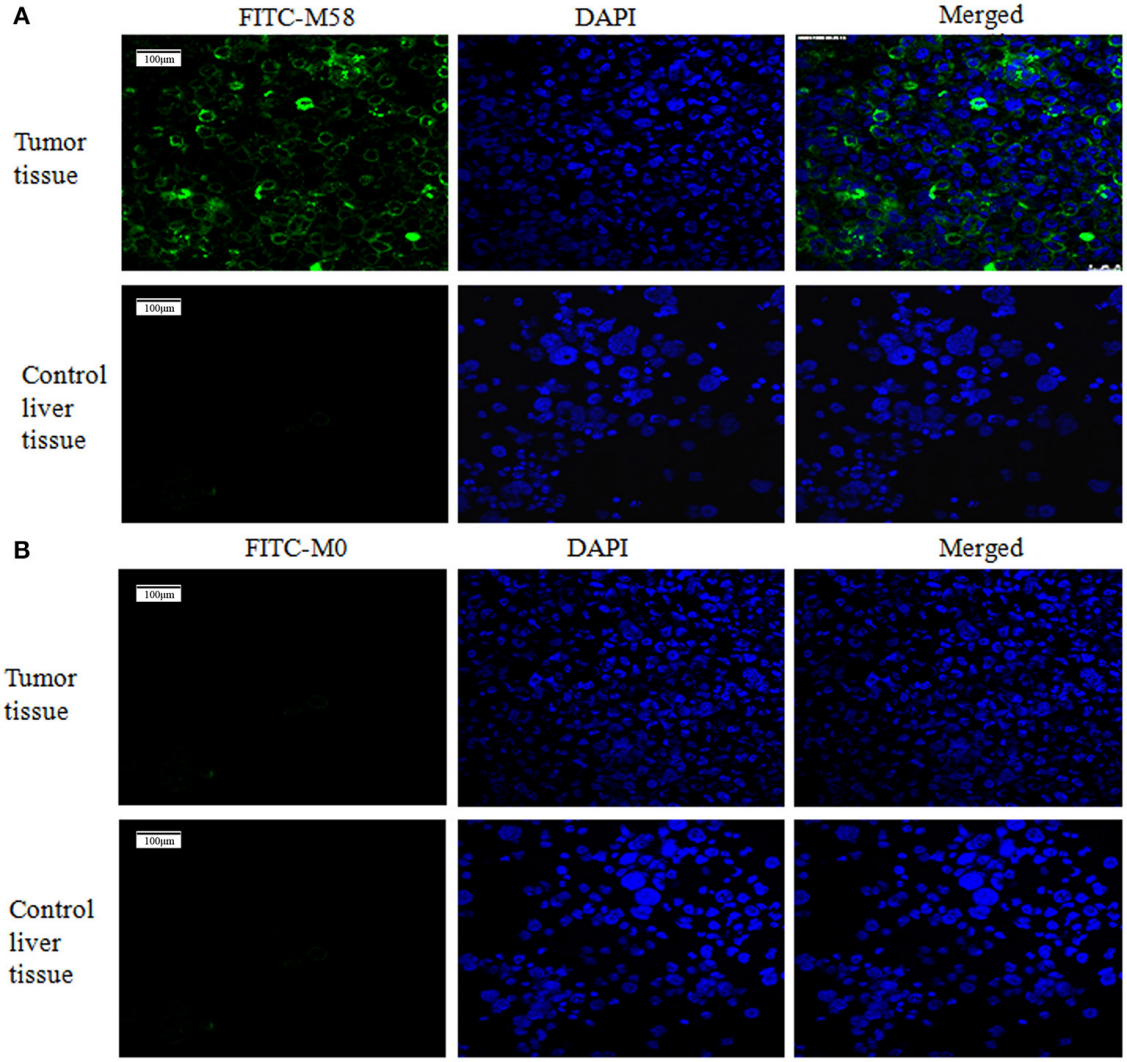
Binding status of polypeptide M58 in the nude BALB/c mice. **(A)** Polypeptide M58 labeled with fluorescein isothiocyanate (FITC) was observed to specifically bind to tumor tissue, but not to control liver tissue. **(B)** The FITC-labeled control polypeptide M0 was observed to bind to neither tumor tissue nor control liver tissue.

Firstly, the palpable tumor nodules appeared 2 weeks after tumor injection and quickly increased to about 10–20 mm in diameter in the following 4 weeks, as shown in Figure 5. The nude mice were randomly divided into two groups (named group M58 and group M0) on average; then they were injected with polypeptide M58 and M0 labeled with FITC, respectively, into the vein of nude mice and dissected to observe the polypeptide distribution in liver tissue after 2 h. Polypeptide M58 labeled with FITC could be visibly observed in tumor tissue but not observed in control liver tissue of the nude BALB/c mice (Figures 6A,B).

## Discussion

Breast cancer is considered the most frequent cancer diagnosed among women worldwide (13). According to Globocan, the estimated incidence of breast cancer for 2018 was 2,088,849 new cases all over the world (14). Breast cancer is a highly heterogeneous disease (15). The major tumor subtypes, hormone receptor (HR) positive, HER2-enriched, and triple negative, are classified based on the immunohistochemical expression of ERs, PRs, and HER2 overexpression or amplification. The immunohistochemical results were the most important and basic bases for the individualized treatments for validating tumor heterogeneity of breast cancer. Triple-negative breast cancer is known as a type of breast cancer lacking expression of ER, PR, and HER2, which are also characterized by aggressive behavior and being prone to local recurrence and distant organ metastasis, as well as poorer survival (16).

The aggressiveness of TNBC and its resistance to standard drug therapies may be related to the presence of BCSCs (17, 18). In all kinds of human cancers, including breast cancer, there is a small subset of CSCs, which are characterized by self-renewal, differentiation, and tumor initiation and development (3). In BCSCs, CD44 antigen (CD44^+^)/signal transducer (CD24^−/*low*^) has been isolated and defined as a recognized phenotype, which may be related to resistance to chemotherapy and/or radiotherapy (19). ALDH1 has been used to identify BCSCs as an alternate cell surface marker (20); meanwhile, the BCSCs that expressed both CD44^+^/CD24^−/*low*^ and ALDH1^+^ had a stronger ability to develop tumors in mice. However, only 1% of ALDH1^+^ BCSCs could simultaneously express the CD44^+^/CD24^−/*low*^ phenotype (21). Therefore, we chose CD44^+^/CD24^−/*low*^ and ALDH1^+^ as biomarker phenotypes and detected their expression status by flow cytometry. In our study, the proportions of CD44^+^/CD24^−/*low*^ and ALDH^+^ for MDA-MB-231 BCSCs were, respectively, 70.5 and 79.3%, while the proportions for common breast cancer cells were very low. The obvious difference between them strongly proved that we have successfully enriched BCSCs and ensured the accuracy in following the experiments. In our enrichment process for BCSCs, we used the “serum and serum-free alternation” culture method, which has been named dual-subtract biopanning, to enrich BCSCs. This method can reduce the disadvantage of the serum-free culture method, which could not enrich enough stem cells, and has minimal damage to stem cells.

Phage display technology involves the expression of sequences of interest inserted within a gene encoding a viral capsid protein, and a modified target peptide is subsequently displayed on the viral capsid of the phage (22). Phage display technology has developed tremendously and changed several fields, such as oncology, cell biology, immunology, pharmacology, and drug discovery (23). Thus, PD is an important technology adopted to solve traditional pharmacologic problems through the discovery of a novel potential target spot or new potential drugs. Several researchers had used PD to screen breast cancer cells and obtained some binding peptides, such as aFGF-binding peptide called AP8 (10), novel peptides that specifically bind with CD44 (11), peptide LS-7 (LQNAPRS)-specific CD133-binding ligand (24), and potential highly specific HER2-binding peptides (25). In the early work of our team, Liu et al. (26) obtained the peptide specific to BCSCs and derived the phage sequence; however, they did not synthesize the polypeptides according to sequence results and did not verify its specificity *in vitro* and *in vivo*. In this study, we discovered eight positive phage clones from PD screening to MDA-MB-231 BCSCs. We chose one positive sequence “TMHYKGTAASES,” which appeared twice from all positive results used for the follow-up experiment. The polypeptide was synthesized according to positive phage sequence, named as M58 and labeled with FITC.

During the verification process *in vitro*, polypeptide M58 labeled with FITC was identified to be specific for MDA-MB-231 BCSCs but not observed in ordinary MDA-MB-231 breast cancer cells, as shown in Figures 4A,B. Meanwhile, the control peptide M0 labeled with FITC was neither specific to MDA-MB-231 BCSCs nor ordinary MDA-MB-231 breast cancer cells. Finally, we established a breast cancer model in nude mice, and then we injected polypeptide M58 and control peptide M0 labeled with FITC into different groups of nude mice. We subsequently observed the distribution of the polypeptides in different tissues under a microscope. We found that polypeptide M58 labeled with FITC was rich in the tumor tissue but poor in the control liver tissue; meanwhile, the control peptide M0 labeled with FITC was observed neither in tumor tissue nor in control liver tissue.

In theory, very few BCSCs can successfully establish mouse xenograft. During the course of nude mouse xenograft, the BCSCs with the concentration of 1 × 10^5^/ml were injected, with the main reason to retain part of BCSCs after mouse xenograft had been established successfully. In addition, nude mice without liver metastasis were used for the identification step *in vivo*.

In conclusion, the MDA-MB-231 BCSCs were successfully enriched with the culture method of “serum and serum-free alternation,” and the stemness was verified with CD44^+^/CD24^−/*low*^ and ALDH^+^ as the biomarker phenotypes by flow cytometry. Then, positive phage sequences that specifically bound to MDA-MB-231 BCSCs were identified from a PD random peptide library. Additionally, one positive phage sequence was selected, and polypeptide M58 was synthesized with the control of peptide M0. At last, the specificity of polypeptide M58 to MDA-MB-231 BCSCs was identified *in vitro* and *in vivo*. Therefore, these results may have broad prospects in the treatment of TNBC or discovery of new target spots for intractable TNBC or as a foundation for novel drugs for TNBC.

## Data Availability Statement

The original contributions presented in the study are included in the article/supplementary material, further inquiries can be directed to the corresponding author/s.

## Ethics Statement

The animal study was reviewed and approved by The Ethics Committee of Shandong Cancer Hospital and Institute.

## Author Contributions

TL: completion of study and writing of manuscript. HW: completion of study. ZL, JZ, and CZ: cells experiment *in vitro*. YL and LZ: animal experiment *in vivo*. FL: writing of manuscript. CH: modification of manuscript. BL: project design. All authors contributed to the article and approved the submitted version.

## Conflict of Interest

The authors declare that the research was conducted in the absence of any commercial or financial relationships that could be construed as a potential conflict of interest.
